# Nanostructure‐Mediated Photothermal Effect for Reinforcing Physical Killing Activity of Nanorod Arrays

**DOI:** 10.1002/advs.202411997

**Published:** 2024-11-18

**Authors:** Guannan Zhang, Zehao Li, Menlin Sun, Ying Lu, Jianbo Song, Wangping Duan, Xiaobo Huang, Ruiqiang Hang, Xiaohong Yao, Paul K Chu, Xiangyu Zhang

**Affiliations:** ^1^ Shanxi Bethune Hospital Shanxi Academy of Medical Sciences Third Hospital of Shanxi Medical University Tongji Shanxi Hospital Taiyuan 030032 China; ^2^ Shanxi Provincial Key Laboratory for Translational Nuclear Medicine and Precision Protection Taiyuan 030006 China; ^3^ Shanxi Key Laboratory of Biomedical Metal Materials College of Materials Science and Engineering Taiyuan University of Technology Taiyuan 030024 China; ^4^ Shanxi Key Laboratory of Bone and Soft Tissue Injury Repair Department of Orthopedics Second Hospital of Shanxi Medical University Taiyuan 030001 China; ^5^ Department of Physics Department of Materials Science and Engineering, and Department of Biomedical Engineering City University of Hong Kong Tat Chee Avenue, Kowloon Hong Kong 999077 China; ^6^ College of Biomedical Engineering Taiyuan University of Technology Taiyuan 030024 China

**Keywords:** anti‐biofilm, light trapping, osseointegration, photothermal therapy, physical puncture

## Abstract

The physical killing of bacteria based on surface topography has attracted much attention due to the sustainable and safe prevention of biofilm formation. However, the antibacterial efficiency of biomedical implants derived solely from nanostructures or microstructures is insufficient to combat bacteria against common infections, such as *methicillin‐resistant Staphylococcus aureus* with thick cell walls. Herein, photothermal therapy is carried out in the presence of nanorod arrays to mitigate infection of biomedical implants. Different from traditional photothermal therapy relying on a photosensitizer, the photothermal effect is mediated by light traps rendered by the nanorod arrays, and consequently, the photosensitizer is not needed. Finite element simulations and experiments are performed to elucidate the light‐to‐thermal conversion mechanism. This photothermal platform, in conjunction with thermosensitive nitric oxide therapy, is applied to treat titanium implant infection. The nanostructure‐mediated photothermal effect destroys bacterial cell walls by inhibiting peptidoglycan synthesis and increasing the membrane permeability by affecting fatty acid synthesis. Furthermore, the nanorods synergistically puncture the bacterial membrane easily as demonstrated by experiments and transcriptome analysis. The results provide insights into the development of efficient antibacterial treatment of implants by combining nanostructures and photothermal therapy.

## Graphical abstract

A novel antibacterial strategy utilizes nanostructured “light traps” mediated PTT to enhance the physical puncture of bacteria by nanorod arrays, effectively eliminating biofilm infections without requiring additional photosensitivities. Combined with nitric oxide therapy, this method effectively prevents MRSA infection on the surface of titanium implants, providing a promising approach for sustainable antibacterial implant surfaces.

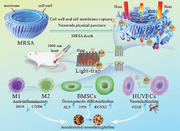

## 1 Introduction

Despite significant advancements in the development of artificial bone implants, infections resulting from bacterial biofilms continue to pose a major challenge to implant longevity and functionality.^[^ ^1^ ^]^ In the conventional management of implant‐related infections, the inappropriate use of antibiotics can compromise the patient's immune system and lead to numerous side effects, notably the emergence of drug‐resistant bacteria.^[^ ^2^ ^]^ The discovery that the special surface morphology of cicada wings, moths, dragonflies, and geckos can induce bacterial cell rupture and death has spurred research on using surface topography to combat bacterial infections.^[^ ^3^ ^]^ Compared to other antibacterial methods, eradicating bacteria by physical interactions not only enhances safety and durability but also mitigates the risk of drug resistance.^[^ ^4^ ^]^ However, a significant time duration is normally required to eliminate bacteria adhering to the surface.^[^ ^5^ ^]^ Additionally, the bacterialcell wall influences the antibacterial efficacy.^[^ ^6^ ^]^ For example, gram‐positive bacteria with thicker cell walls, such as *methicillin‐resistant Staphylococcus aureus* (MSRA) which cause implant‐related infections, cannot be killed easily by this mechanism alone.^[^ ^3a^ ^]^

Photothermal therapy (PTT) has garnered significant attention in the eradication of biofilms due to its broad‐spectrum antibacterial efficacy, minimally invasive nature, and ability to circumvent drug‐resistant strains.^[^ ^7^ ^]^ The elevated temperature generated by PTT induces irreversible damage to bacterial cell walls and membranes and produces synergistic effects in combination with antibiotics, reactive oxygen (ROS), and nitric oxide (NO) to kill bacteria.^[^ ^8^ ^]^ PTT requires photosensitizers to convert light energy into heat.^[^ ^9^ ^]^ Although photosensitizers such as carbon nitride, red phosphorus, black phosphorus, and molybdenum disulfide show antibacterial effects, the long‐term structural stability and biosafety of implants need to be validated clinically.^[^ ^10^ ^]^ In addition, the photothermal conversion efficiency decreases if the second near‐infrared (NIR‐II) window (1000‐1700 nm) with a higher tissue penetration depth is adopted.^[^ ^9^ ^]^ Consequently, it is essential to develop PTT technology that can be activated by NIR‐II light without the need for photosensitizers.

**Scheme 1:**
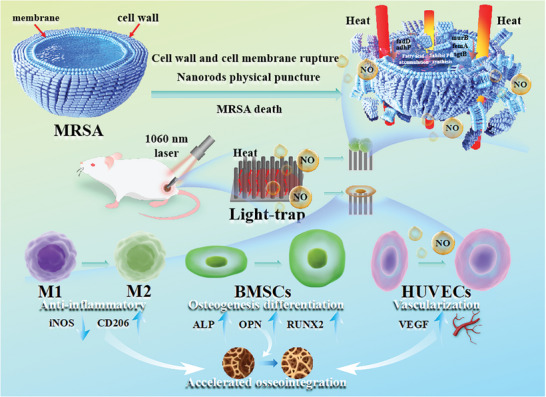
Schematic illustration of TiO_2_/SNO nanorod arrays for biofilm eradication and bone regeneration.

The photothermal properties of materials primarily depend on their light absorption capacity and photothermal conversion efficiency.^[^ ^11^ ^]^ In general, bulk materials have limited ability to absorb light. To enhance light absorption, various microstructures, and nanostructures have been proposed to increase light reflection, refraction, and scattering in order that the photon energy can be trapped by the nanostructure to minimize loss before it is released as thermal energy.^[^ ^11^, ^12^, ^13^, ^14^ ^]^ It is thus desirable to develop nanostructures with enhanced NIR‐II light absorption by light trapping to kill bacteria. Herein, theoretical calculations are first performed to predict the impact of the diameter, height, and angle of light incidence on the photothermal properties of nanostructures. Based on the calculated results, titanium dioxide (TiO_2_) nanorod arrays with light trapping capability are fabricated on titanium (Ti). To achieve enhanced antibacterial activity at a relatively mild temperature while regulating the immune microenvironment and promoting osseointegration, a thermosensitive NO donor (S‐nitrosuccinic acid, SNO) is grafted onto the nanorod arrays (TiO_2_/SNO).^[^ ^8^, ^15^ ^]^ Upon NIR‐II light irradiation, the nanostructure‐mediated photothermal effect destroys the bacterial cell wall and membrane by affecting the synthesis of peptidoglycan and fatty acids, respectively, in addition to enhancing physical protrusion of the nanorod arrays into bacteria to achieve safe and efficient elimination of *Staphylococcus aureus* (*S. aureus*) biofilm. The nanorod arrays mitigate immune response, accelerate tissue repair, promote angiogenesis in human umbilical vein endothelial cells (HUVECs), enhance osteogenic differentiation of bone marrow mesenchymal stem cells (BMSCs), and foster osseointegration by mild‐temperature stimulation **Scheme** **1**.

## 2 Results and Discussion

### 2.1 Theoretical Photothermal Calculations

The photothermal conversion can be categorized into three mechanisms: thermal vibrations of molecules, non‐radiative relaxation of electron‐hole pairs, and plasmonic heating, and light absorption also has a significant impact on light‐to‐heat conversion.^[^ ^11^ ^]^ In addition to the inherent absorption properties of materials, the design and fabrication of photo‐capturing structures can change the direction of light propagation to enhance internal reflection and light absorption.^[^ ^11^, ^12^ ^]^ Common light‐capturing structures include arrays, porous structures, and particles.^[^ ^12^, ^16^ ^]^ The thermal, optical, and electronic properties of nanomaterials can be tailored by manipulating their shape, size, composition, and surrounding environment.^[^ ^17^ ^]^ Herein, numerical simulation is carried out to predict the effects of different structural parameters on the optical response.^[^ ^18^ ^]^ The mechanical puncturing effects of nanostructures on the bacterial cell wall are also taken into account.^[^ ^19^ ^]^

**Figure 1:**
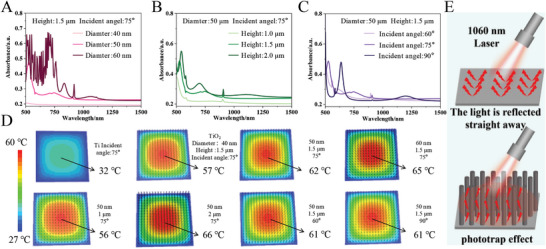
Theoretical calculations: A) Simulation of absorption spectra for different nanorod diameters, B) Heights, and C) Incidence angles; D) Simulation of thermal distributions for different nanorod diameters, heights, and incidence angles; E) Schematic diagram illustrating the photo trapping effect by the TiO_2_ nanorod arrays.

With increasing nanorod diameters (40, 50, and 60 nm) and heights (1, 1.5, and 2 µm), the optical absorption coefficients increase (**Figure** **1**A,B). When the diameter of TiO_2_ nanorod increases to 60 nm (height: 1.5 µm, incident angle: 75°), or when the height reaches 2 µm (diameter: 50 nm, incident angle: 75°), the surface temperatures can rise to 65 and 66 °C, respectively (Figure 1D). In contrast, the surface temperature of TiO_2_ without nanostructure only increases to 32 °C. However, the change of the light incidence angle (60°, 75°, and 90°) has little effect on the light absorption coefficient of the nanorod array (Figure 1C), and no significant differences in surface heating are observed (Figure 1D). In the NIR range, the photothermal properties of TiO_2_ depend on the inherent dielectric properties of the materials rather than local plasmonic heating and non‐radiative relaxation of electron‐hole pairs.^[^ ^3c^ ^]^ The properly designed nanostructures can trap light and enhance reflection, refraction, and scattering to improve the photothermal effects.

### 2.2 Characterization and Photothermal Properties

**Figure 2:**
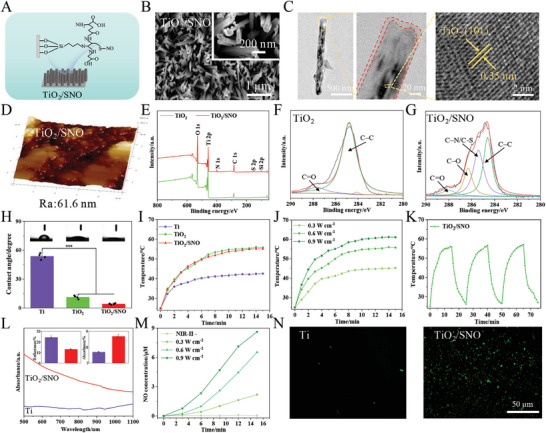
A) Schematic diagram of the TiO_2_/SNO nanorod arrays on Ti; B) Field‐emission scanning electron microscopy (FE‐SEM) images, C) Transmission electron microscopy (TEM) images, and D) Atomic force microscopy (AFM) images of TiO_2_/SNO; E) XPS survey spectra of TiO_2_ and TiO_2_/SNO; F) XPS C 1*s* spectra of TiO_2_ and G) TiO_2_/SNO; H) Contact angles on Ti, TiO_2_, and TiO_2_/SNO; I) Thermograms of Ti, TiO_2_, and TiO_2_/SNO after irradiation; J) Thermograms of TiO_2_/SNO at different powers by the 1060 nm laser; K) TiO_2_/SNO heating and cooling curves for 3 cycles; L) Absorption spectra; M) NO release from TiO_2_/SNO during 1060 nm laser irradiation; N) Fluorescence staining images of NO generated.

Under the guidance of theoretical calculations, the construction of TiO_2_ nanorod arrays fully considered the regulation of photothermal performance through variations in diameter and height, as well as its inherent physical puncturing effect on bacteria. While maintaining the photothermal efficiency, nanorods with smaller diameters exhibit an enhanced puncturing effect on bacteria. Ultimately, we prepared a nanorod arrays with a diameter of 50 nm and a height of 1.5 µm. Since a high temperature can damage adjacent normal tissues, a thermosensitive NO donor is grafted onto the nanorods. In addition to NO release under hyperthermia to kill bacteria at a mild temperature, NO can modulate the immune microenvironment and promote angiogenesis and osseointegration. As shown in Figure S1 (Supporting Information), the TiO_2_ array with a nanorod diameter of 50 nm and length of 1.5 µm is successfully fabricated on Ti hydrothermally, and grafting of SNO does not change the nanorod morphology (**Figure** **2**B), although the roughness decreases from 118 to 61.6 nm (Figure 2D). Figure 2C shows the presence of an inorganic layer (between the red dotted lines) on the TiO_2_/SNO array, confirming modification by SNO. The array of TiO_2_ exhibits the (101) planes with a lattice spacing of 0.35 nm. Figure S3 (Supporting Information) reveals no significant difference in the X‐ray diffraction (XRD) spectra between TiO_2_ and TiO_2_/SNO. They show diffraction peaks at 54.1° and 70.2° corresponding to the rutile phase of TiO_2_, and the crystal structure is not affected by the introduction of SNO.

The X‐ray photoelectron spectroscopy (XPS) spectra in Figure 2E–G reveal C 1*s*, O 1*s*, and Ti 2*p* peaks in TiO_2_ and TiO_2_/SNO, and the N 1*s*, S2p, and Si 2*p* peak emerges from TiO_2_/SNO after silanization. As shown in Table S3 (Supporting Information), after silanization of TiO_2_, the content of Si increased from 0% to 4.83%. And after grafting SNO, the content of Si decreased to 2.06% and the content of S increased to 3.14%. As shown in Figure 2F, the peaks at 284.6 and 287.7 eV arise from C─C and C═O. After silanization and SNO covalent binding, the peaks of C─N/C─S and C─O can be observed at 285.4 and 286.3 eV (Figure 2G), indicating successful SNO modification.^[^ ^17^ ^]^ The N═O peak at 1562 cm^−1^ detected by Fourier Transform Infrared Spectroscopy (FTIR) confirms the modification with SNO (Figure S4, Supporting Information).^[^ ^19a^ ^]^ The hydrophilicity is presented in Figure 2H. The alkali thermal reaction enhances the hydrophilicity of Ti with the contact angle decreasing from 54.5° to 13.4°. The contact angle further decreases to 5.2° after modification with SNO, which may be attributed to changes in the surface morphology and chemistry.

The temperature variation during irradiation by the 1060 nm laser in the Phosphate Buffered Saline (PBS) solution (1 mL) is monitored to evaluate the photothermal properties of different samples. As shown in Figure 2I, the surface temperature is positively correlated with the irradiation time and reaches a plateau after ≈10 min. Under identical irradiation conditions, TiO_2_ and TiO_2_/SNO show larger temperature increase than Ti, but the difference between TiO_2_ and TiO_2_/SNO is not significant, suggesting that the modification with SNO has a limited impact on the photothermal conversion efficiency. The photothermal conversion efficiency of TiO_2_/SNO is calculated to be up to 39.2% through the fitting curve analysis (Figure S7A, Supporting Information). Additionally, the temperature increase also depends on the laser power. The temperature of TiO_2_/SNO increases from 45.3 to 61.1 °C as the laser power is increased (Figure 2J). The temperature rise and fall curves of TiO_2_/SNO after three cycles of laser irradiation and shutdown are shown in Figure 2K. Remarkably, there is no significant deviation between subsequent cycles and initial irradiation, indicating excellent photothermal stability of TiO_2_/SNO and promising potential for repeated phototherapy. Figure 2L indicates that TiO_2_/SNO with a nanorod structure exhibits higher absorption across all bands than Ti. Moreover, TiO_2_/SNO has a bigger absorption rate and lower reflectivity than Ti. In summary, the theoretical and experimental results are consistent.

As shown in Figure 2M,N, the ability of the TiO_2_/SNO phototherapy to release NO is verified. Figure 2M illustrates that the release of NO is directly proportional to both the laser power and illumination time. Under NIR‐II laser irradiation, this system enables the controlled release of NO. Specifically, when the power of 1060 nm laser power increases, the concentration of NO rises from 2.1 to 8.6 µm due to the higher surface temperature, consequently increasing the release of heat‐sensitive SNO. The generation of NO under NIR‐II irradiation by TiO_2_/SNO is corroborated by the fluorescence probe (Figure 2N). After laser irradiation, distinct NO fluorescence staining is visible on the surface of TiO_2_/SNO, while only minimal NO fluorescence is detected from Ti, suggesting that TiO_2_/SNO produces NO during laser irradiation.

### 2.3 Antibacterial Effects In Vitro

Since nanorod arrays only exert limited physical puncturing effects on bacteria with thick cell walls, we aim to enhance the antibacterial effects by nanostructure‐mediated PTT disruption of their cell wall and membranes. However, PTT at a mild temperature only causes limited bacterial damage. As shown in Figure S9 (Supporting Information), the nanorod arrays eliminate *S. aureus* biofilms at a temperature of 60 °C which can damage adjacent normal tissues.^[^ ^20^ ^]^ Song et al. have discovered that NO can suppress bacterial heatshockprotein70 (HSP70, A heat shock protein that helps bacteria cope with stress situations such as high temperature, hypoxia, chemicals, etc.) expression, increase bacterial susceptibility to heat, and improve the therapeutic effectiveness of PTT.^[^ ^21^ ^]^ Therefore, nanorod‐mediated PTT is combined with a thermosensitive NO donor.

**Figure 3:**
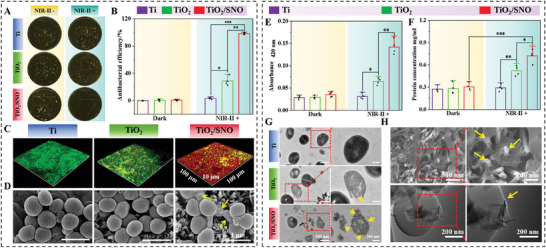
Antibiofilm activity: A) Photographs of the coated plates; B) Antibacterial rates; C) Live/dead staining fluorescence images of the biofilms; D) SEM images of bacterial; E) Hydrolysis of ONPG and F) Protein leakage of bacterial; G) TEM images of bacterial; H) SEM and TEM images of bacterial punctured by nanorods.

The spread plate method was used to evaluate the efficacy of eliminating *S. aureus* biofilms (**Figure** **3**A) and to calculate the antibacterial rate (Figure 3B). As expected, TiO_2_/SNO effectively eliminates biofilms under NIR‐II laser irradiation with an antibacterial rate of 99.3%. Live/dead fluorescence staining further proves the anti‐biofilm effect of TiO_2_/SNO under NIR‐II laser irradiation (Figure 3C). As the irradiation time increases, the bacteria in the biofilm die gradually, and the biofilm is essentially eliminated after 15 min (Figure S10, Supporting Information).

The SEM was used to examine the morphology of the *S. aureus* biofilm (Figure 3D). After illumination, the bacteria with dense surface of Ti and TiO_2_ showed a typical spherical shape, and the cell membrane was intact. In contrast, the TiO_2_/SNO surface shows reduced biofilm coverage, accompanied by severe atrophy and distortion in some bacteria. The Ortho‐nitrophenyl‐β‐galactoside (ONPG) hydrolysis assay is employed to evaluate the permeability of the bacterial cell membranes.^[^ ^22^ ^]^ As shown in Figure 3E, no significant hydrolysis of ONPG is without laser irradiation. In contrast, NIR‐II laser irradiation increases the hydrolysis efficiency of ONPG of TiO_2_/SNO. Protein leakage within bacteria (Figure 3F) is in line with the trend of ONPG hydrolysis due to extensive nutrient loss caused by severe damage to the bacterial cell membranes. The results show that nanorod array‐mediated NIR‐II phototherapy has enhanced bactericidal effects by destroying bacterial cell walls and membranes. SEM and TEM reveal strong interactions between the bacteria and TiO_2_/SNO nanorods after phototherapy, and the bacteria collapse around the nanorods (Figure 3G,H). Prominent nanorods (indicated by yellow arrows) are visible on the bacteria, suggesting that those bacteria are killed ultimately by penetration of their compromised cell walls and membranes by the nanorods.

The mechanism of *S. aureus* biofilm elimination by TiO_2_/SNO under NIR‐II light irradiation is proposed. Initially, upon NIR‐II light irradiation, TiO_2_/SNO exhibits the light trapping effects leading to localized overheating and consequent damage to the bacterial cell walls and membranes. The subsequent temperature triggers the production of NO from SNO. NO enhances the photothermal effect and destroys the cell walls and membranes. Finally, the nanorods physically puncture the compromised cell walls to produce intracellular nutrient leakage and bacterial death.

### 2.4 Transcriptome Sequence Analysis and Antibacterial Mechanism

**Figure 4:**
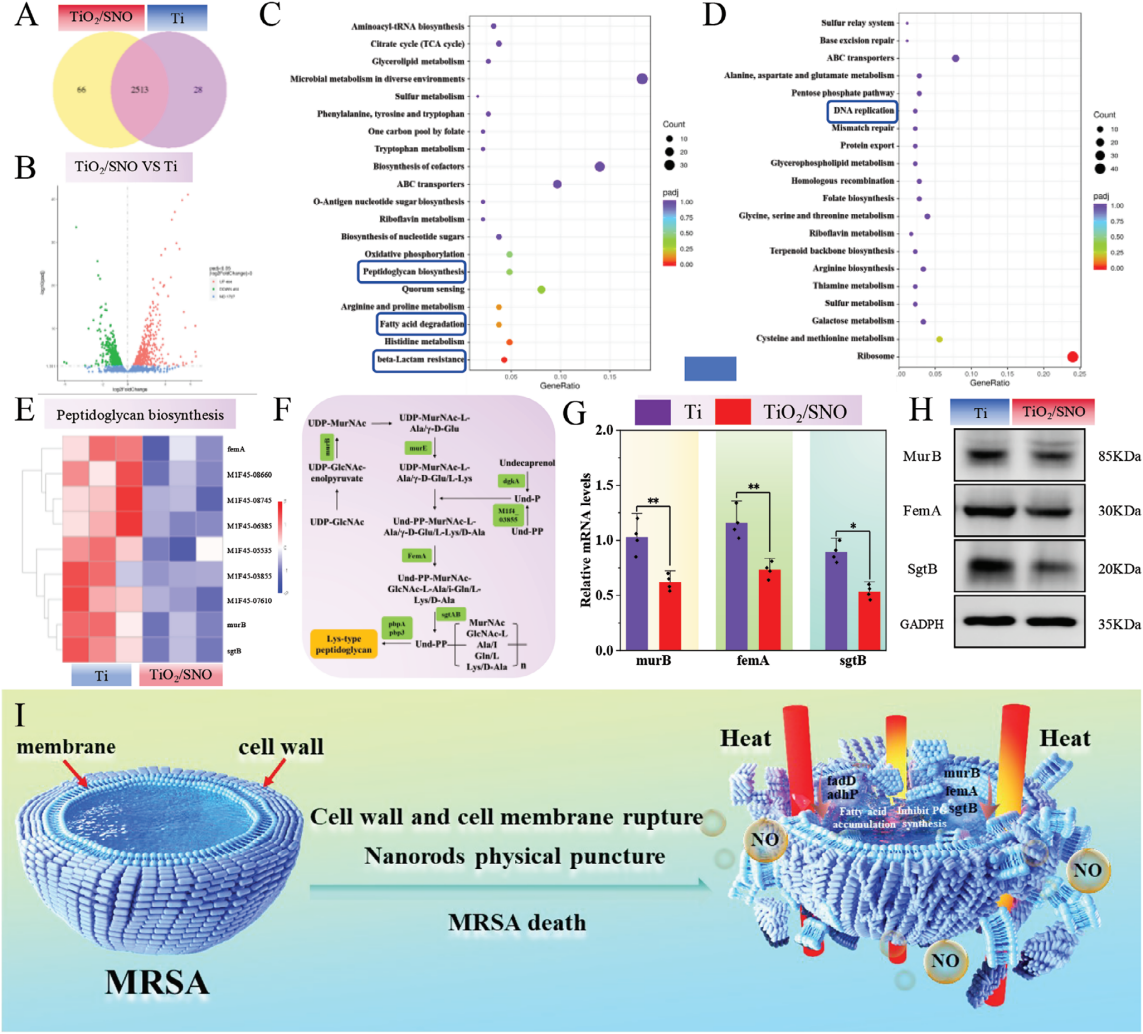
Changes of MRSA transcriptome in the Ti and TiO_2_/SNO groups after phototherapy: A) Venn diagram of the number of DEGs; B) Volcano maps of the DEGs distribution; C) Downregulated and D) Upregulated DEGs enriched in the KEGG pathway; E) Heat map of Lys‐type peptidoglycan genes; F) Synthesis pathways of Lys‐type peptidoglycan; G) Expression levels of murB, femA, and sgtB genes by RT‐PCR; H) Western blot results of different protein expressions; I) Mechanism of bacterial death upon NIR‐II light irradiation.

RNA transcriptome sequencing is conducted on MRSA to investigate the antibacterial mechanism of TiO_2_/SNO. 2513 genes are co‐expressed in Ti and TiO_2_/SNO groups, but only 28 and 66 genes are expressed by the Ti and TiO_2_/SNO groups, respectively (**Figure** **4**A). Figure 4B presents the gene expression volcano map of TiO_2_/SNO showing 484 up‐regulated and 450 down‐regulated genes compared to the Ti group, suggesting that the gene expression of MRSA is notably affected by TiO_2_/SNO upon 1060 nm laser irradiation. The differentially expressed genes (DEGs) are analyzed by the Kyoto Encyclopedia of Genes and Genomes (KEGG). As shown by KEGG down in Figure 4C, down‐regulated genes are mainly enriched in the pathways of peptidoglycan biosynthesis, fatty acid degradation, and beta‐Lactam resistance. KEGG up is shown in Figure 4D, and up‐regulated genes are mainly concentrated in pathways such as Cysteine and methionine metabolism and ribosomes.

After confirming the gene enrichment pathways, the relevant pathways are analyzed to elucidate the antimicrobial mechanism of TiO_2_/SNO. As the primary constituent in the bacterial cell wall, peptide glycan (PG) plays a crucial role in stabilizing the cell membrane, withstanding the high intracellular osmotic pressure, and regulating bacterial growth.^[^ ^23^ ^]^ The Gram‐positive cell wall possesses a PG layer with a thickness ranging from 30 to 100 nm, which is ≈4–5 times thicker than that of Gram‐negative bacteria.^[^ ^24^ ^]^ This larger thickness made it more effective in resisting external physical penetration. Figure 4E,F presents the pathways for Lys‐type PG synthesis, and the relevant genes (murB, femA, sgtB) are down‐regulated. The murB, femA, and sgtB genes encode the synthesis of UDP‐N‐acetylenolpyruvoylglucosamine, glycine glycyltransferase FemA, and monofunctional peptidoglycan glycosyltransferase SgtB, respectively. The downregulation of murB impacts the second step in peptidoglycan biosynthesis, while the downregulation of femA reduces cross‐linking of peptidoglycan within the bacterial cell wall.^[^ ^25^ ^]^ Additionally, the downregulation of sgtB impedes the transfer of sugar groups to peptidoglycan monomers during bacterial cell wall synthesis, thus preventing the formation of linear peptidoglycan chains.^[^ ^26^ ^]^ RT‐PCR and western blot are performed to confirm the expressions of relevant genes and proteins. As shown in Figure 4G,H, the expressions of relevant genes and proteins involved in PG synthesis are significantly downregulated for the TiO_2_/SNO group, suggesting that the MRSA cell walls suffers damage.

Figure S11A,B (Supporting Information) illustrates the fatty acid degradation pathway and gene expressions of MRSA of the TiO_2_/SNO group. All the genes in the image are down‐regulated, suggesting the accumulation of fatty acids in MRSA. The presence of excess fatty acids implies a higher membrane permeability because TiO_2_/SNO disrupts the bacterial cell membrane, consistent with the ONPG and protein leakage assays.^[^ ^27^ ^]^ The primary way MRSA develops drug resistance is through its high tolerance to beta‐lactam analogs.^[^ ^28^ ^]^ Figure S11 (Supporting Information) illustrates the genes associated with β‐lactam resistance. The TiO_2_/SNO group has lower expression levels of these genes than the Ti group. Hence, TiO_2_/SNO reduces bacterial drug resistance and increases the efficacy of bacterial eradication. In summary, under 1060 nm laser irradiation, TiO_2_/SNO destroys cell wall and membrane of MRSA, enhances the physical puncturing effect of the nanorods on bacteria, reduces the endocannabinoid resistance of MRSA, and kills bacteria.

### 2.5 Anti‐Biofilm and Immune Response In Vivo

**Figure 5:**
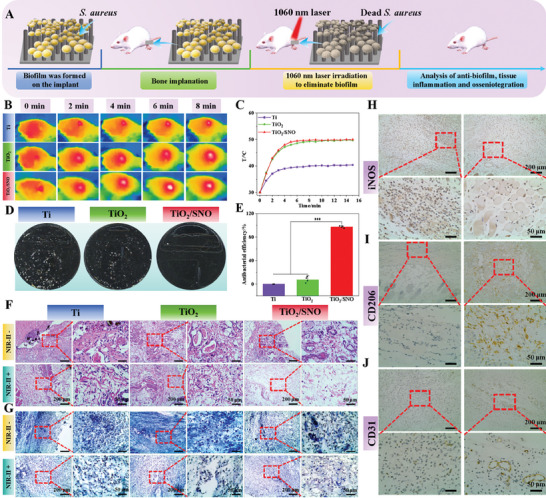
In vivo evaluation of anti‐biofilm and immune responses: A) Schematic diagram of in vivo experimental design with the anti‐biofilm model; B) In vivo thermogram of the TiO_2_/SNO implant under 1060 nm laser irradiation for 15 min; C) Temperature curve; D) Photographs of the coated plates of TiO_2_/SNO in vivo; and E) Antibacterial rates; F) H&E and G) Giemsa staining images of tissues around the implants; H) iNOS, I) CD206, and J) CD31 immunohistochemistry of tissues around the implants.

The anti‐biofilm effect in vivo was evaluated by irradiation of *S. aureus* biofilm‐covered implants in the tibia of rats with NIR‐II light. The real‐time temperature of the implants is monitored by a thermal imager in vivo. As shown in **Figure** **5**B,C, the temperature of TiO_2_ and TiO_2_/SNO increases rapidly from 30 °C to ≈50 °C in 6 min before stabilizing. And the temperature of Ti increases to only 38 °C. These findings suggest that the NIR‐II laser can penetrate tissues to generate heat underneath.

The samples are removed two days after surgery, and the bacteria remaining are isolated ultrasonically. Figure 5D,E reveals a substantial number of colonies on the surfaces of both Ti and TiO_2_. Conversely, the TiO_2_/SNO exhibits only sporadic colonies with an antibacterial rate of 97.7%. Laser irradiation and physical puncturing produce synergistic effects.

After exogenous bacterial infection of the implant, inflammatory cells quickly accumulate at the sites of infection, and excessive inflammation can produce implant failure ultimately.^[^ ^8a^ ^]^ Bacterial infection and inflammation in the tissues surrounding the implant are observed by Giemsa and H&E staining, respectively. As shown in Figure 5F,G, a notable presence of neutrophils is observed from the tissue surrounding the implant without phototherapy, suggesting severe infection. Despite phototherapy, both the Ti and TiO_2_ groups are infected. In contrast, the TiO_2_/SNO group shows fewer inflammatory cells and residual bacteria, reflecting the excellent anti‐biofilm capability in vivo.

The regulatory effects of TiO_2_/SNO on inflammation are investigated by co‐culturing macrophages and bacteria. Figure S13 (Supporting Information) shows that the TiO_2_/SNO group experiences a significantly higher uptake of bacteria by macrophages under NIR‐II light irradiation compared to the other groups, suggesting that in the early stage of infection, the TiO_2_/SNO group enhances cellular phagocytosis by promoting macrophage polarization toward the pro‐inflammatory M1 phenotype and rapidly eliminates bacteria. Subsequent immunohistochemistry analysis of the surrounding tissue after 3 days shows that the expression of the proinflammatory factor iNOS decreases, while the anti‐inflammatory factor CD206 increases for TiO_2_/SNO compared to Ti (Figure 5H,I). These findings provide evidence that TiO_2_/SNO phototherapy mitigates inflammation produced by bacterial infection. In addition, the angiogenic potential of the TiO_2_/SNO group is superior to that of the Ti group based on the CD31 immunohistochemistry analysis (Figure 5J). The enhancement may result from the photoinduced production of NO by TiO_2_/SNO under NIR‐II light irradiation and subsequent promoted expression of vascular endothelial growth factor (VEGF).

### 2.6 Metabolomics Analysis and Repair Mechanism After Eliminating Bacterial Infection by Phototherapy

**Figure 6:**
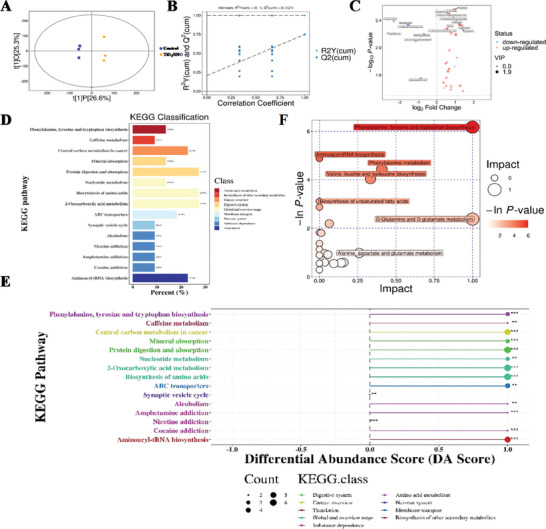
Metabolomics changes of related tissues after anti‐biofilm infection treated by phototherapy of the Ti and TiO_2_/SNO groups: A) Scatter plot of OPLS‐DA scores; B) OPLS‐DA model image; C) Volcano image; D) KEGG pathway classification diagram; E) Differential Abundance Score map; F) KEGG pathway bubble plot char.

The effects of TiO_2_/SNO are investigated by metabolomics analysis. The horizontal axis of the scatter plot in the OPLS‐DA results indicates the between‐group differences and the vertical axis exhibits the within‐group differences. Larger horizontal distances between samples reflect greater between‐group differences, while closer vertical distances indicate better within‐group reproducibility (**Figure** **6**A). The results show that the samples are all within the confidence interval thus validating the data. The corresponding OPLS‐DA model plots demonstrate a satisfactory fit of the test model and also indicate statistical significance (Figure 6B). The volcano plots show up‐regulation of immunomodulatory and signaling‐related differential metabolites, as well as down‐regulation of inflammatory mediators, suggesting a significant difference between the Ti and TiO_2_/SNO groups (Figure 6C).

The KEGG pathway classification graph reflects the percentage of different types of differential metabolites. For example, amino acid metabolism and amino acid biosynthesis account for 40.91%, biosynthesis of other secondary metabolites accounted for 9.09%, nucleotide metabolism for 13.64%, 2‐oxocarboxylic acid metabolism for 27.27%, aminocarbamoyl‐tRNA biosynthesis for 22.73%, and ABC transporters accounted for 18.18% (Figure 6D). The ABC transporter transports nutrients and 2‐oxocarboxylic acid metabolism produces energy for the biosynthesis of substances. The findings demonstrate that TiO_2_/SNO effectively facilitates tissue regeneration following phototherapy.

The differential abundance score plots disclose the general trends for specific metabolites for the TiO_2_/SNO group (Figure 6E). The up‐regulation of 2‐oxocarboxylic acid metabolism, ABC transporters, tyrosine and tryptophan biosynthesis, and phenylalanine indicate accelerated metabolism and generation of more energy.^[^ ^29^ ^]^ The up‐regulation of mineral absorption, protein digestion, and absorption shows that the organism can recover from the damage, which is reflected at the genetic level by accelerated nucleotide metabolism and aminoacyl‐tRNA biosynthesis.^[^ ^30^ ^]^ These results show that compared to Ti, TiO_2_/SNO effectively eliminates biofilm infection and accelerates the repair of organisms after phototherapy.

Figure 6F shows the KEGG pathway bubbles that are enriched with metabolites. The biosynthesis of phenylalanine, tyrosine, and tryptophan and the metabolism of phenylalanine participate in glucose metabolism and lipid metabolism. In addition, some energy is released from the iodinated synthesis of unsaturated fatty acids. The biosynthetic processes of valine, leucine, and isoleucine are associated with protein synthesis. The metabolism of D‐glutamate and D‐glutamine is also implicated in protein synthesis and energy‐related processes, while the activation of glutamine by NO generated from the materials further contributes to the functional role.^[^ ^31^ ^]^ The metabolism of alanine, aspartate, and glutamate promotes RNA synthesis and participates in amino acid anabolism, thereby upregulating aminoacyl‐tRNA biosynthesis. The metabolic changes indicate that the organisms are in the damage repair phase and more energy is required for RNA and protein synthesis, leading to a significant up‐regulation of the energy‐related processes and the excellent bactericidal ability of TiO_2_/SNO.

Overall, TiO_2_/SNO effectively eradicates biofilm infection and mitigates the associated inflammatory response in infected rats after phototherapy. Subsequently, the damage sustained during infection is repaired, thus requiring more energy to support RNA and protein synthesis. Consequently, more energy is acquired by nutrient absorption and accelerated energy production. The metabonomic results demonstrate that TiO_2_/SNO exhibits remarkable efficacy in eradicating biofilm infections, mitigating inflammatory responses, and expediting tissue repair after phototherapy.

### 2.7 Osseointegration Capacity of TiO2/SNO

Angiogenesis is essential for bone integration at the bone‐implant interface, so the in vitro angiogenesis capacity of HUVECs is assessed.^[^ ^8a^ ^]^ As shown in Figure S15 (Supporting Information), the cells on Ti, TiO_2_, and TiO_2_/SNO proliferate with time with or without laser irradiation besides no noticeable cytotoxicity. Upon irradiation with a 0.3 W cm^−2^ laser, TiO_2_/SNO facilitates the proliferation of HUVECs by generating small quantities of NO. However, when the laser power is raised to 0.6 W cm^−2^, cell atrophy occurs due to the high temperature, leading to some cell mortality (Figure S16, Supporting Information). The secretion of VEGF serves as an indicator of the activity of HUVECs. The ELISA method is employed to quantitatively evaluate the levels of VEGF secreted by HUVECs. As shown in Figure S17 (Supporting Information), consistent with live‐dead fluorescence staining, only TiO_2_/SNO enhances VEGF secretion by HUVECs under laser irradiation. Previous studies have shown that NO diffusion in the vascular system plays a role in vascular permeability and influences the long‐term response of HUVECs in terms of survival, migration, and proliferation. Additionally, NO upregulates the VEGF expression and promotes angiogenesis. The well‐developed vascular network supplies osteoblasts and osteoinductive growth factors with essential nutrients and oxygen for osseointegration.^[^ ^8a^ ^]^ Numerous studies have demonstrated that nanostructures can regulate angiogenesis through various factors, including shape, size, charge, hydrophobicity, and surface functional groups. However, TiO_2_ and TiO_2_/SNO with a nanorod structure do not enhance endothelial cell proliferation or VEGF secretion under dark conditions. During NIR‐II laser irradiation, TiO_2_ also fail to exhibit any promotional effects. In contrast, only TiO_2_/SNO is found to stimulate VEGF secretion during NIR‐II laser irradiation. Consequently, we conclude that the influence of the nanorod structure on angiogenesis is not the primary factor in this investigation.

**Figure 7:**
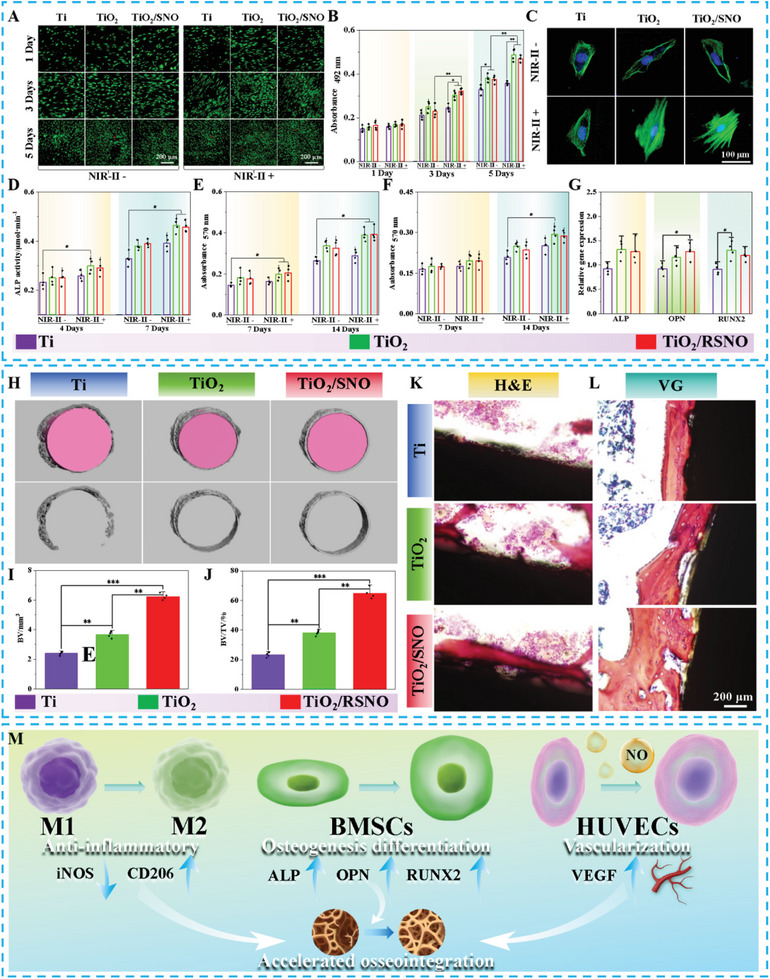
A) Live/dead fluorescence staining images and B) MTT cell proliferation results of BMSCs cells; C) Fluorescence pictures of BMSCs; D) Quantitative ALP activity, E) Collagen secretion, and F) ECM mineralization of BMSCs; G) Expression of osteogenic genes; H) 3D images of the reconstructed implant by micro‐CT; I) Quantitative analysis of BV and J) BV/TV; K) H&E and L) Van Gieson's picro fuchsin stained images of bone tissue around Ti, TiO_2_, and TiO_2_/SNO; M) Schematic diagram of TiO_2_/SNO promoting osseointegration.

BMSCs, as osteoblast precursor cells, play a pivotal role in bone regeneration.^[^ ^32^ ^]^ The fluorescence images by live/dead cell staining of BMSCs are presented in **Figure** **7**A. The number of cells in all the samples increases monotonically with time, and no dead cells are observed with or without laser irradiation. The cell proliferation rates of TiO_2_ and TiO_2_/SNO are higher than that of Ti, suggesting that SNO modification does not influence cell proliferation. Laser irradiation at 0.3 W cm^−2^ stimulates cell proliferation in all the samples. However, at a higher laser power of 0.6 W cm^−2^, the higher temperature and NO production give rise to cell death in the TiO_2_/SNO group (Figure S18, Supporting Information). Figure 7B shows the MTT results of BMSCs, which are basically consistent with live/dead cell fluorescence staining. In the absence of laser irradiation, the spreading of cells on the TiO_2_ and TiO_2_/SNO surfaces is larger than that on the Ti surface. The cells have an elongated morphology, indicating that the nanostructures contribute to enhanced cell spreading. Upon exposure to NIR‐II light (0.3 W cm^−2^), the cells on the Ti surface show slightly more spreading. However, a significant increase in cell spreading is observed from both TiO_2_ and TiO_2_/SNO in addition to abundant filopodia formation. The results suggest that controlled mild hyperthermia enhances the adhesion and spreading of BMSCs.

The osteogenic differentiation of BMSCs is assessed by monitoring the Alkaline phosphatase (ALP) activity, collagen secretion, and extracellular matrix (ECM) mineralization in the early and late stages, respectively. As shown in Figure 7D–F, the ALP activity, collagen secretion, and ECM mineralization levels of BMSCs on the TiO_2_ and TiO_2_/SNO surfaces are higher than those on Ti at different time times. Under NIR‐II light irradiation (0.3 W cm^−2^), these indicators are further improved, indicating that nanostructure and mild hyperthermia promote osteogenic differentiation synergistically.

RT‐QPCR is performed to detect the expression levels of ALP, OPN, and RUNX2 genes. As shown in Figure 7G, after culturing for 7 days in the osteoinduction medium, the expression levels of the related osteogenic genes in BMSCs of TiO_2_ and TiO_2_/SNO are higher than that of Ti. There is no significant difference between TiO_2_ and TiO_2_/SNO, indicating that the upregulation of osteogenic genes is primarily attributable to the influence of the nanostructure, while the modification with SNO does not pose a significant effect.

The nanoscale morphology of implants impacts cell behavior, as shown by the appropriate TiO_2_ nanomorphology that supports cell growth. Nanostructured surfaces can enhance integrin expression, local adhesion, and the development of cytoskeletal structures.^[^ ^33^ ^]^ Nanostructured scaffolds have been shown to trigger osteogenesis through the BMP receptor signaling pathway.^[^ ^29^ ^]^ The study conducted by Wang et al. demonstrated that photothermal stimulation can enhance the expression of heat shock proteins (HSP 47 and HSP 70) and expedite bone defect healing through the promotion of type I collagen biosynthesis.^[^ ^34^ ^]^ Zhang et al. have shown that mild local heating (40–43 °C) during NIR radiation increases the expressions of Hsp47 and BMP2.^[^ ^35^ ^]^ Our research results indicate that samples with nanorod morphology can enhance osteogenic differentiation, a process that is further facilitated by mild NIR‐II photothermal stimulation. This suggests that the combination of both factors can promote the osteogenic differentiation of BMSCs.

The in vivo bone integration on TiO_2_/SNO is investigated. Infection may hinder the osseointegration of implants, and it is necessary to monitor the formation of new bone tissues around the *S. aureus* biofilm‐covered implants after phototherapy. Figure 7H shows the 3D bone tissue image around the implant by micro‐CT scanning (gray indicating the new bone tissue and pink representing the implant). Quantitative analysis (Figure 7I,J) reveals that the bone volume (BV) and bone volume/tissue volume (BV/TV) of Ti, TiO_2_, and TiO_2_/SNO are 2.36, 3.68, 6.34, and 22.6%, 37.5%, 65.8%, respectively. Moreover, the mineralized bone tissue (indicated in red) at the interface between the bone and the implant is examined by H&E staining and Van Gieson's picro fuchsin staining. As shown in Figure 7K,L, a limited quantity of new bone is identified surrounding the Ti implant, with a slight increase around the TiO_2_ implant, but a significant amount of new bone is observed from the TiO_2_/SNO implant. These results demonstrate that TiO_2_/SNO counteracts bacterial infection and enhances bone integration after phototherapy. The biosafety of TiO_2_/SNO is assessed by H&E staining of the heart, liver, spleen, lung, and kidney. Figure S21 (Supporting Information) reveals no significant abnormality or damage confirming the biological safety of TiO_2_/SNO.

TiO_2_/SNO phototherapy eradicates biofilm infection as verified by our in vitro and in vivo experiments. Although the production of heat and NO during bacterial removal has a small negative impact on the viability of normal cells, the regenerative properties mitigate serious tissue damage caused by brief phototherapy.^[^ ^32^ ^]^ Furthermore, as the temperature decreases to ≈42 °C, this side effect diminishes gradually and can even promote cell proliferation and functional expression under mild hyperthermia. In summary, TiO_2_/SNO offers an effective approach in biofilm eradication based on hyperthermia‐induced physical puncturing of bacteria by nanorods, while simultaneously offering desirable effects such as promoting bone integration by immune response inhibition, angiogenesis promotion, and osteogenic differentiation enhancement.

## 3 Conclusion

TiO_2_ nanorod arrays are prepared on Ti hydrothermally. NIR‐II photothermal therapy is conducted by taking advantage of synergistic physical puncturing of the bacterial cell walls and membranes by the nanoarray structure to kill bacteria. The light‐to‐thermal conversion mechanism is related to light trapping by the nanoarrays due to the multiple interactions of light, including reflection, refraction, and scattering. Without needing a photosensitizer, the photothermal platform combining thermosensitive NO therapy effectively eliminates biofilms on Ti implants. Photothermal stimulation destroys the bacterial cell walls and membranes, and the nanorods puncture and damage the bacteria easily. Moreover, phototherapy attenuates the immune response, facilitates angiogenesis and osteogenic differentiation, and augments osseointegration. The light‐to‐thermal conversion mechanism overcomes the drawbacks of traditional photothermal therapy which requires a photosensitizer. The results reveal a new research direction for photothermal therapy in the biomedical field.

## 4 Experimental Section

### Preparation of TiO_2_/SNO

The medical Ti foil was cut into 10 mm × 10 mm pieces. It was then treated hydrothermally at in 1 m NaOH (220 °C, 4 h), and sonicated for 1 min. The sample was treated hydrothermally again in 0.5 m NaOH (220 °C, 2 h) and sonicated for 1 min. It was soaked in 0.5 m HCl for 30 min to replace Na^+^ and annealed (600 °C, 2 h) to yield the TiO_2_ nanorod array. To covalently attach the SNO to the sample surface, TiO_2_ was immersed in a 4% (v/v) aminopropyltriethoxysilane/anhydrous ethanol (≥98%, Merck) solution for silylation for 12 h, and the silylated sample was immersed in the SNO solution (5 × 10^−2^ m, ≥97%, Sigma) at 30 °C for 6 h to obtain TiO_2_/SNO.

### Characterization

FE‐SEM (Ultra55, Zeiss, Germany) and TEM (Tecnai G20, FEI, USA) were used to observe the surface morphology of the nanorod arrays. The surface roughness of the sample was measured by AFM (XE‐100, Park Systems), and the crystal structure was determined by XRD (Rigaku Dmax‐3C, Cu K_α_ radiation) and TEM. The elemental composition was measured by XPS (K‐Alpha, Thermo Fisher Scientific), and the surface contact angles were measured by the JC2000D2 (Powereach, China). The 46 Solidspec‐3700 spectrophotometer UV‐2 (Shimadu, Japan) was used to acquire the NIR absorption and reflection spectra.

### Photothermal Characteristics In Vitro

The samples were immersed in 1 mL of deionized water and then irradiated with a 1060 nm laser (0.3–0.9 W cm^−2^) for 15 min. The sample surface temperature was by infrared thermography at 1 min intervals.

### Detection of NO

The concentration of NO produced by TiO_2_/SNO under 1060 nm laser (0.3–0.9 W cm^−2^) irradiation was tested by the NO kit (Cat#BC1475, Solarbio, China).

### Antibacterial Properties

The bacteria used in the experiment were purchased from China Center of Industrial Culture Collection, CICC. The antibacterial activity was determined with *S. aureus* and MRSA by the plate counting method. The samples were sterilized with 75% medical alcohol for 10 min. Then the samples were incubated in 2 mL of diluted bacterial suspension (1 × 10^9^ CFU mL^−1^) to form complete biofilms on the surface. The samples covered with biofilms were placed in PBS (1 mL) and irradiated with the 1060 nm laser (0.5 W cm^−2^) for 15 min. The samples were then sonicated for 10 min to separate the bacteria. The resulting bacterial solution was diluted by a factor of 1000, and a volume of 50 µL was dispensed on a petri dish. The bacteria were incubated for 24 h at 37 °C and the colonies were counted. The antibacterial rate was determined by the following equation: Antibacterial rate = (β_control_−β_sample_)/β_control_ × 100%. Here, the average numbers of colonies in the control and sample groups were labeled β_control_ and β_sample_, respectively.

The efficacy of laser irradiation in removing biofilms was assessed by AO/PI fluorescent staining (Cat#abs9727, Absin, China). The mixed dye (50 µL) was introduced for staining for 20 min. After rinsing with PBS, the samples on the slides were inverted and confocal scanning laser microscope (CLSM) was performed in three random areas. The biofilm was fixed with glutaraldehyde (GA, 3%, aladdin, China), dehydrated with gradient alcohol, and gold‐coated. Three random areas were examined by FE‐SEM.

### Bacterial Membrane Permeability

The permeability of the *S. aureus* biofilm was evaluated using ONPG as a probe. The optical density (420 nm) of the supernatant of samples was determined by the ONPG detection kit (cat# ST429, Beyotime, China).

### Protein Leakage

The bacterial suspension (1 mL, 5 × 10^7^ CFU mL^−1^) treated with laser irradiation was collected and centrifuged (4 °C, 5000 rpm, 10 min), and the BCA kit (cat# P0009, Beyotime, China) was used to determine the protein concentration.

### Transcriptome Analysis

MRSA was cultured in TiO_2_/SNO and irradiated with a 1060 nm laser for 15 min. During the logarithmic growth phase, bacteria were collected via cryogenic centrifugation and immediately frozen in liquid nitrogen. Each experimental group was repeated three times under the same conditions. Total RNA extraction and cDNA library preparation were conducted on the bacteria, followed by sequencing performed by Novogene (Beijing, China). Expression data were analyzed using DESeq2 (Version 1.24.0) software to identify differentially expressed genes (DEGs), with the criteria set to |log_2_FC|≥1 and a p‐value <0.05. The biological functions and pathways of these DEGs were investigated using the KEGG database, and KEGG pathway analysis was carried out using KOBAS software. Fisher's exact test was employed to assess the significance of the pathways in order to elucidate gene functional differences between the samples.

### Gene Expression

The biofilm was collected by centrifugation after being irradiated by 1060 nm laser. Total bacterial RNA was extracted from biofilms using a bacterial RNA extraction kit (Vazyme, China). Then, the Color reverse transcription kit (EZBioscience, USA) and 2 × Color SYBR Green qPCR master mix (EZBioscience, USA) were used to perform RT‐PCR experiments. RT‐PCR was employed to determine the relative expression levels of energy metabolism pathway genes with 16S rRNA as the internal reference gene. The primer sequences are shown in Table S1 (Supporting Information).

### Cell Culture

HUVECs and BMSCs were used in the in vitro cell experiments using culture media of DMEM and α‐MEM supplemented with sodium bicarbonate (2.2 g L^−1^), streptomycin (0.133 g L^−1^), penicillin (0.0667 g L^−1^), and 10% fetal bovine serum (FBS, Sijiqing, China). The cells were cultured in a chamber (37 °C, 5% CO_2_), and the seeding density was 2 × 10^4^ cells cm^−2^. The samples were sterilized with 75% medical alcohol for 10 min.

### Live/Dead Fluorescence Staining

Live/Dead fluorescence staining (0.5 µL mL^−1^ calcein AM and 2 µL mL^−1^ ethidium homodimer, Invitrogen, USA) was used to determine the toxicity of different samples to HUVECs and BMSCs. After 1, 3, and 5 days of cell culture on the surface of samples, the samples were gently washed with PBS. The stain (50 µL) was dropped onto the sample surface (1 h) and observed by CLSM.

### VEGF Secretion of HUVECs

The culture supernatant of HUVECs was collected after culturing for 24 h, and the VEGF ELISA kit (Abcam, UK) was used to quantify the secretion of VEGF.

### Cell Proliferation

The proliferation of BMSCs was quantified by the 3‐(4,5‐dimethylthiazol‐2‐yl)−2,5‐diphenyltetrazolium bromide (MTT, Sigma, USA) colorimetric assay. 900 µL of the medium and 100 µL of MTT (5 mg mL^−1^) were added to each sample. After incubation at room temperature for 4 h, 1 mL of dimethyl sulfoxide (DMSO, Sigma, USA) was added to dissolve the purple crystal, and the absorbance was measured at a wavelength of 492 nm.

### Cytoskeleton

The skeleton assembly assay was performed to evaluate the spreading of cells on the surface. The cells cultured for 24 h were fixed with 1 mL of 3% GA for 1 h and stained with 50 µL of fluorescein isothiocyanate (FITC, Sigma, USA) and 4′,6‐diamidino‐2‐phenylindole (DAPI, Sigma, USA) for 40 and 10 min, respectively. Three random areas were imaged by CLSM.

### ALP Activity

The ALP activity of BMSCs was quantified by the ALP Activity Kit. After differentiation, the lysis solution (60 µL, cat# P0013j, Beyotime, China) was added to lyse the cells, and the absorbance at 405 nm was measured using the ALP activity kit (cat# P0321, Beyotime, China).

### Collagen Secretion and ECM Mineralization

The Sirius Red (Sigma, USA) staining was used to quantify collagen secretion of BMSCs. Similarly, alizarin red (Aladdin, China) staining was used to determine ECM mineralization. After being fixed, the cells were stained with Sirius red (0.1% weight/volume) and alizarin red (40 mm), respectively. Then, the samples were washed with the appropriate solution until colorless. The crystals were dissolved with a combination of NaOH/methanol (1 mL, 0.2 m, 1:1) and cetylpyridinium chloride (500 µL, 10%), and the absorbances of the two solutions at 570 nm were measured.

### Osteogenic‐Related Gene Expression

After culturing for 5 days, the entire intracellular RNA was collected using the RNA isolation kit (Eastep Super Total RNA Extraction Kit). Subsequently, the RNA underwent reverse transcription to generate a 1200 ng DNA system using the transcription kit (PrimeScript RT reagent Kit). The expression levels of the osteogenic genes, ALP, OPN, and RUNX2, were determined on the Quant Studio real‐time fluorescence quantitative PCR instrument. Blank plates with osteoblasts were employed as the control, and the ΔΔCT method was applied to compute the expression levels of the relevant genes, with the GAPDH gene being the normalization control. The primer sequences are shown in Table S2 (Supporting Information).

### In Vivo Evaluation

The animal experiments were approved and recorded by the Animal Ethics Committee of Taiyuan University of Technology.

### Antibacterial Assessment

A rat infection model was established using *S. aureus* to evaluate the effectiveness in preventing bacterial biofilm formation in vivo. Eighteen male Sprague‐Dawley rats weighing between 300 and 320 grams were divided into three experimental groups: Ti, TiO_2_, and TiO_2_/SNO. The samples were sterilized with 75% medical alcohol for 10 min. Then the samples (Φ1.5 mm × 3 mm) were placed in the *S. aureus* suspension (1 mL, 1 × 10^9^ CFU mL^−1^) for two days and the surface was covered with biofilm. Ti rods with existing biofilms were surgically implanted into the tibia of the rats. Following suturing, the wounds were irradiated with the 1060 nm laser irradiation (0.6 W cm^−2^) for 15 min. After two days, three rats from each group were euthanized, and the implants were retrieved for coating plate tests to appraise the intraosseous anti‐biofilm properties. Furthermore, the soft tissues surrounding the implants were fixed in 4% paraformaldehyde (PFA) overnight, dehydrated, cleared, and embedded to prepare the pathological sections. These sections were stained with H&E and Giemsa, and three random areas were examined for bacterial infection and histomorphological changes by a Zeiss microscope (Imager M2, Carl Zeiss, Germany). Immunostaining with iNOS, CD206, and CD31 was also performed to determine the inflammatory response and angiogenesis.

### Metabolomics Analysis

Metabolomics testing was performed by Biotree Biotech Co., Ltd. (Shanghai, China).

### New Bone Formation

The remaining rats were euthanized 30 days after surgery, and the tibial section containing the implant was extracted for hard tissue analysis. The specimens were fixed in 4% PFA for 2 days and dehydrated with gradient alcohol. Micro‐CT imaging was used to observe new bone formation around the implants at a resolution of 10 µm. Various thresholds (σ = 0.8, support = 1, bone threshold = 35, and implant threshold = 211) were applied to distinguish between the bone and titanium implants in generating the 3D images. Subsequently, the samples were embedded in resin, cured, and cut into 50 µm thick hard tissue sections. The sections underwent van Gieson's picro fuchsin staining and H&E staining to assess new bone growth.

### Statistical Analysis

All the experimental results were expressed as mean ± SD with n ≥ 3, and the statistical differences were calculated by the two‐tailed Student's *t*‐test (^***^ *p* < 0.001, ^**^ *p* < 0.01, or ^*^ *p* < 0.05).

## Conflict of Interest

The authors declare no conflict of interest.

## Supporting information



Supporting Information

## Data Availability

The data that support the findings of this study are available from the corresponding author upon reasonable request.
