# Correction to “Nanostructure‐Mediated Photothermal Effect‐Reinforced Physical Killing Activity of Nanorod Arrays”

**DOI:** 10.1002/advs.202520117

**Published:** 2025-11-19

**Authors:** Guannan Zhang, Zehao Li, Menlin Sun, Ying Lu, Jianbo Song, Wangping Duan, Xiaobo Huang, Ruiqiang Hang, Xiaohong Yao, Paul K Chu, Xiangyu Zhang

Adv. Sci. 2025, 12, 2411997

https://doi.org/10.1002/advs.202411997

The authors regret that an incorrect immunofluorescence staining image for Live/dead (green/red) was used for TiO_2_ samples without NIR‐II irradiation at 3 days in Figure S15 (Supporting Information). The modified correct version is shown below. As shown in Figure S15 (Supporting Information), the cells on Ti, TiO_2_, and TiO_2_/SNO proliferate with time with or without laser irradiation besides no noticeable cytotoxicity. Upon irradiation with a 0.3 W cm^−2^ laser, TiO_2_/SNO facilitates the proliferation of HUVECs.

**Figure:**
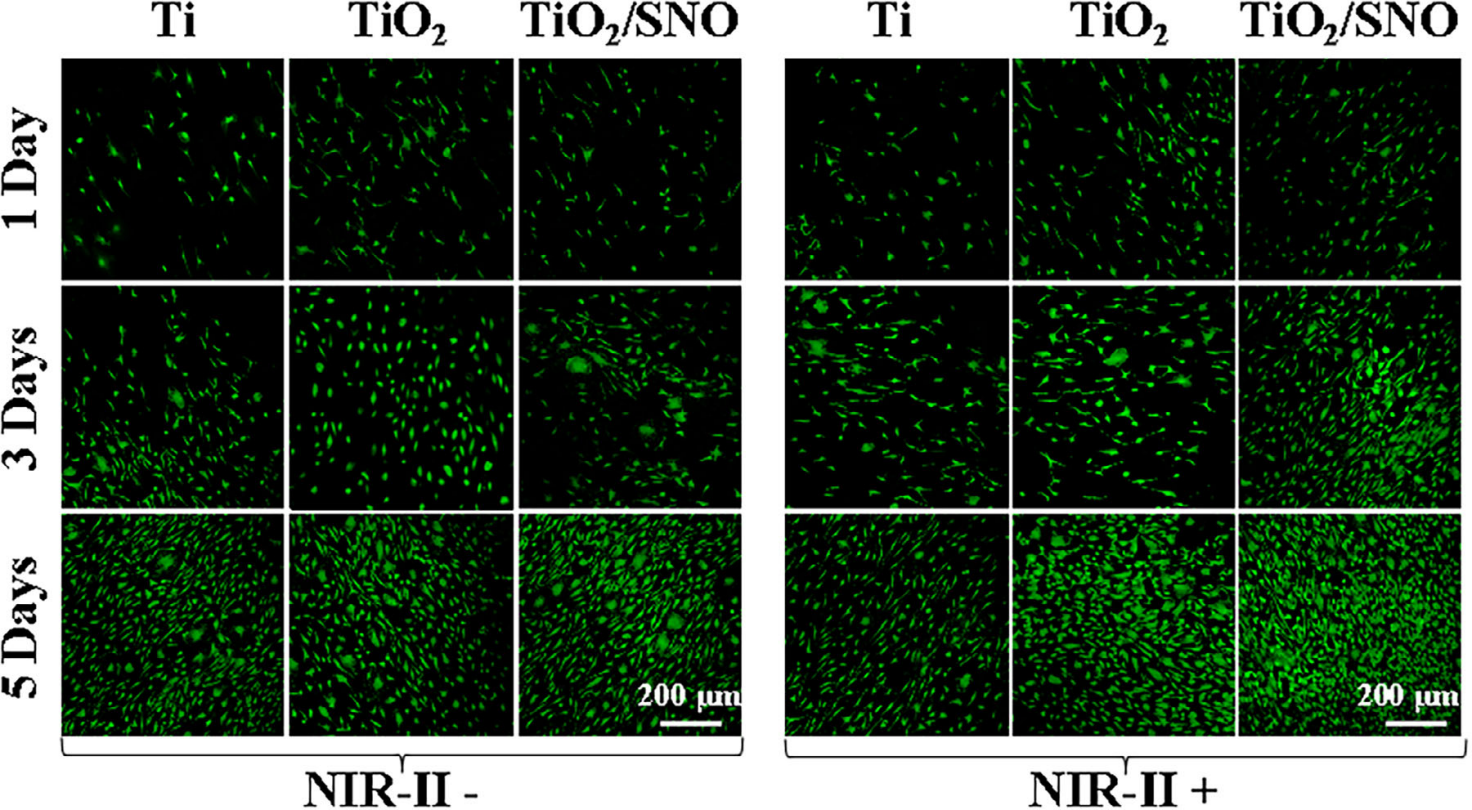
**Figure** **S15**. Live/dead (green/red) fluorescence staining images of HUVECs.

We apologize for this error.

## Supporting information



Supporting Information

