# Supplementary material for: Correction to “Nanostructure‐Mediated Photothermal Effect‐Reinforced Physical Killing Activity of Nanorod Arrays”
Source: Adv Sci (Weinh). 2025 Nov 19;13(1):e20117. doi: 10.1002/advs.202520117 (PMC12767043; doi:10.1002/advs.202520117)
Supplement: Supplementary file 1 — Supporting Information [file ADVS-13-e20117-s001.docx]

**Supplementary Information**

**Nanostructure-mediated** **and photothermally reinforced physical killing activity of nanorod arrays**

Guannan Zhang ^#b,c^, Zehao Li ^#a^, Menlin Sun ^a^, Ying Lu ^b,c^, Jianbo Song* ^b,c^, Wangping Duan ^d^, Xiaobo Huang ^a^, Ruiqiang Hang ^a^, Xiaohong Yao ^a^, Paul K Chu* ^e^, Xiangyu Zhang* ^a,f^

^a^ Shanxi Key Laboratory of Biomedical Metal Materials, College of Materials Science and Engineering, Taiyuan University of Technology, Taiyuan 030024, China

^b^ Shanxi Bethune Hospital, Shanxi Academy of Medical Sciences, Third Hospital of Shanxi Medical University, Tongji Shanxi Hospital, Taiyuan 030032, China

^c^ Shanxi Provincial Key Laboratory for Translational Nuclear Medicine and Precision Protection, Taiyuan 030006, China

^d^ Shanxi Key Laboratory of Bone and Soft Tissue Injury Repair, Department of Orthopedics, Second Hospital of Shanxi Medical University, Taiyuan 030001, China

^e^ Department of Physics, Department of Materials Science and Engineering, and Department of Biomedical Engineering, City University of Hong Kong, Tat Chee Avenue, Kowloon, Hong Kong, China

^f^ College of Biomedical Engineering, Taiyuan University of Technology, Taiyuan 030024, China

# These authors contributed equally

*Corresponding Author: E-mail: [zhangxiangyu@tyut.edu.cn](mailto:zhangxiangyu@tyut.edu.cn) (X Zhang)

*Corresponding Author: E-mail: [paul.chu@cityu.edu.hk](mailto:paul.chu@cityu.edu.hk) (P.K. Chu)

*Corresponding Author: E-mail: [jianbo2611@s](mailto:jianbo2611@126.com)xmu.edu.cn (J Song)

**Experimental Section**

*Macrophages culture:* The murine macrophage cell line RAW 264.7 (TIB-71, ATCC, VA, USA) was used in this study. RAW 264.7 was cultured in DMEM medium containing 1% streptomycin, 1% penicillin, and 10% fetal bovine serum (FBS) before being placed in a cell culture chamber at 37 °C. Lipopolysaccharide (LPS, 100 ng/mL) was added to the medium to form an activation medium.

*S. aureus/macrophage co-culture experiment:* MRSA at a concentration of 1 × 10^8^ CFU/mL was stained with the CFDASE fluorescent probe (cat# C005115, Beyotime, China) for cell proliferation for 20 min. The stained bacteria were mixed with macrophages and treated under different conditions. The mixture was shaken on an oscillator (37 °C, 120 rpm) for 3 h and images were collected using a fluorescence microscope.


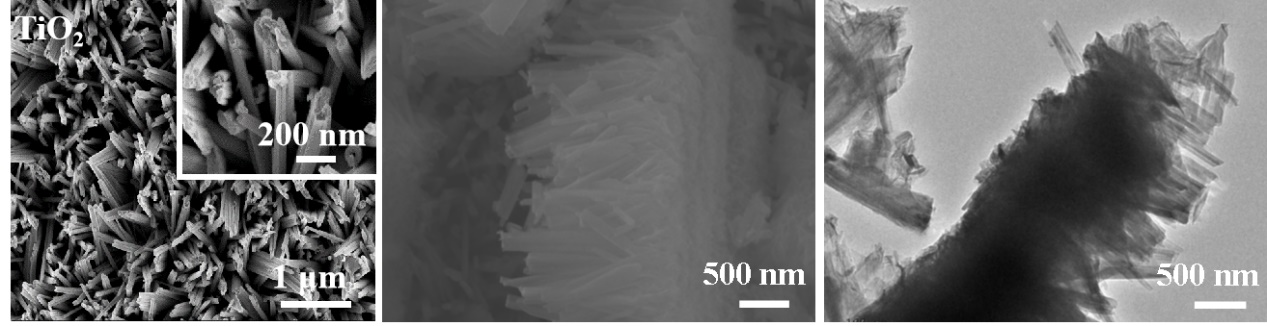


Figure S1. SEM and TEM images of TiO_2_ nanorod arrays.


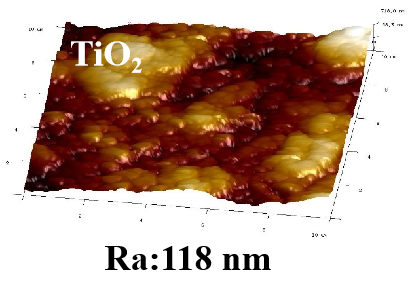


Figure S2. AFM image of TiO_2_ nanorod arrays.


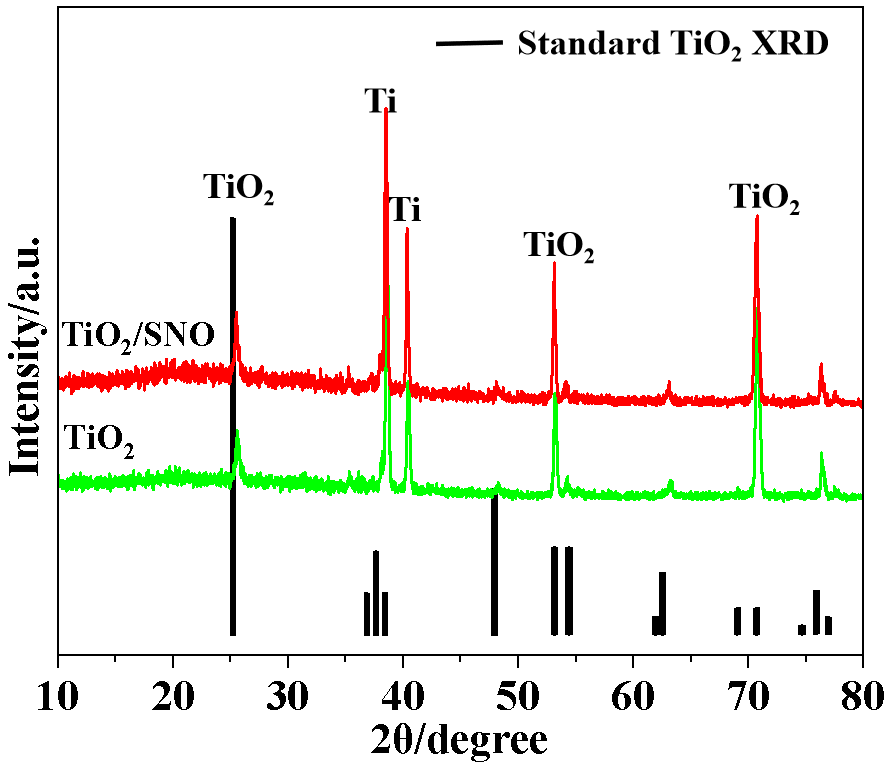


Figure S3. XRD patterns of TiO_2_ and TiO_2_/SNO.


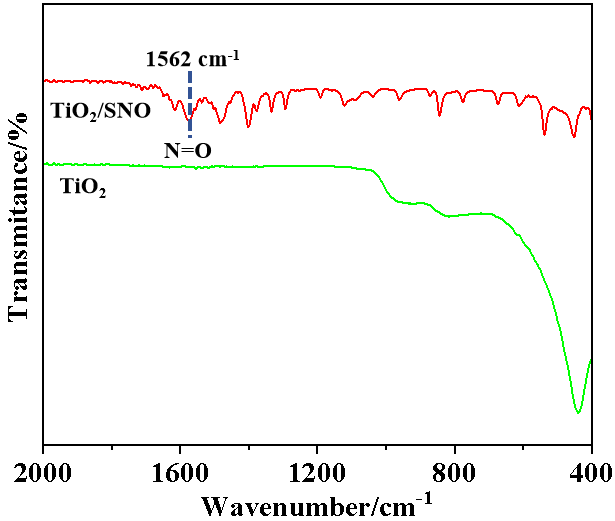


Figure S4. FTIR spectra of TiO_2_ and TiO_2_/SNO.


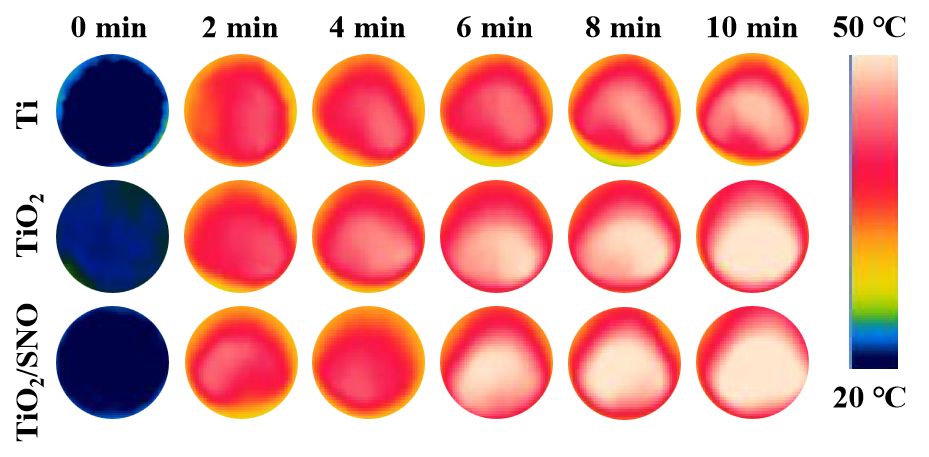


Figure S5. Photothermal images of Ti, TiO_2_, and TiO_2_/SNO at different time points.


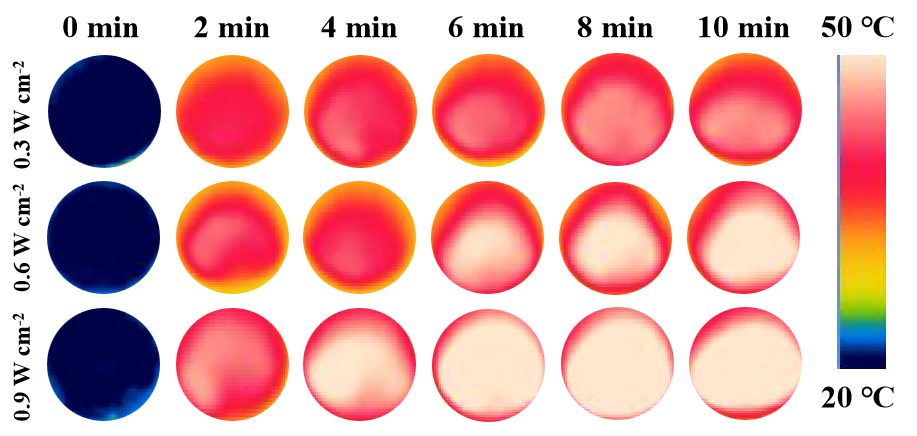


Figure S6. Photothermal images of TiO_2_/SNO for different irradiation power.


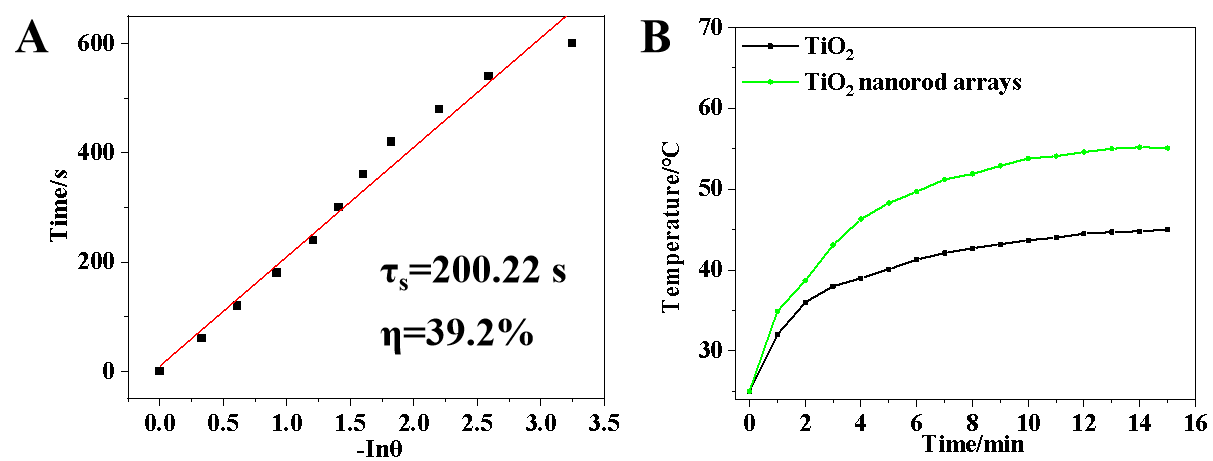


Figure S7. (A) The calculated photothermal conversion efficiency of TiO_2_/SNO; (B) Photothermal heating curves of TiO_2_ and TiO_2_ nanorod arrays under 1060 nm laser irradiation.


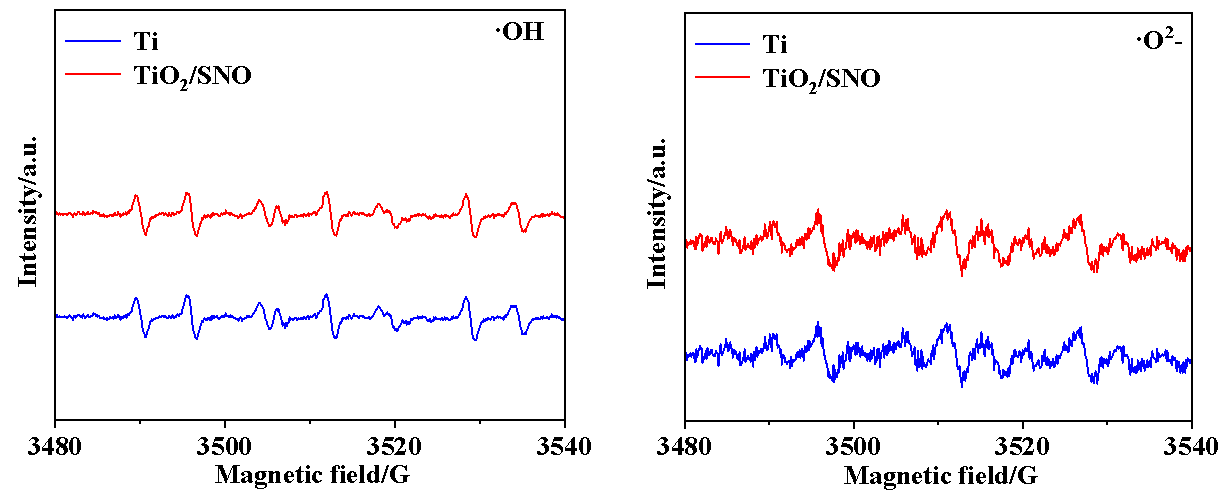


Figure S8. ·OH and ^1^O_2_ detection of Ti and TiO_2_/SNO by ESR with the DMPO probe.


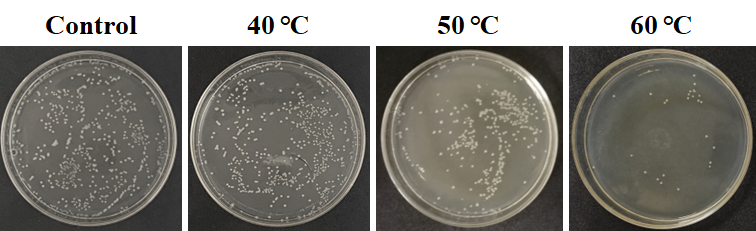


Figure S9. Photographs of coated plates of TiO_2_ nanorod arrays at different temperatures.


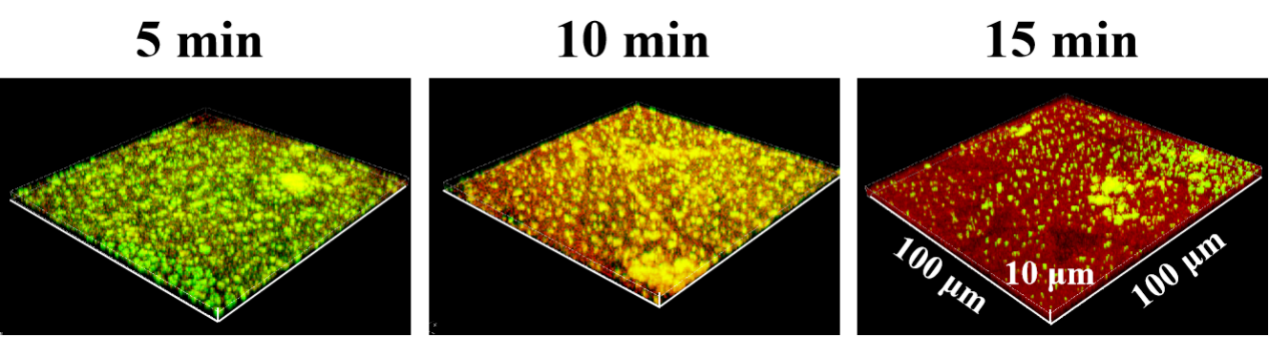


Figure S10. Fluorescence images of *S. aureus* biofilms on TiO_2_/SNO at different time points.


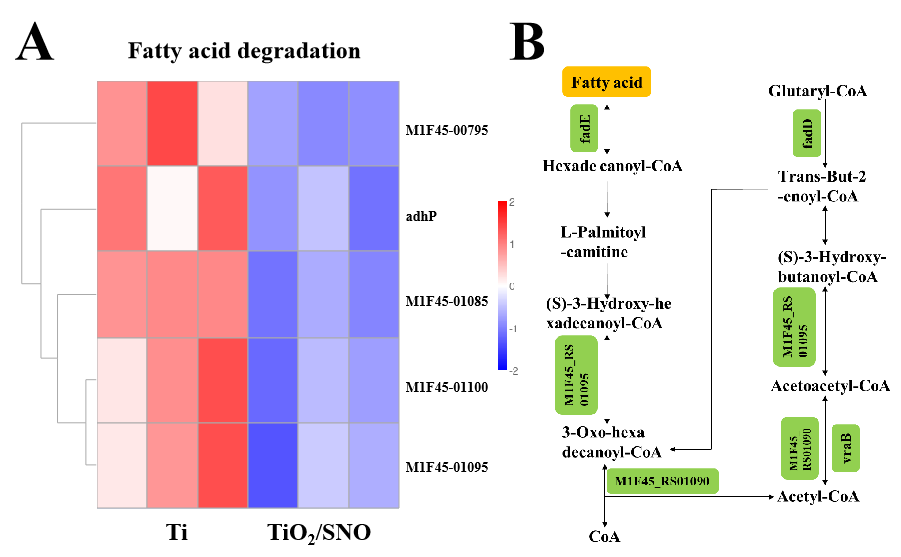


Figure S11. (A) Heat map of fatty acid genes and (B) Degradation pathway of fatty acid.


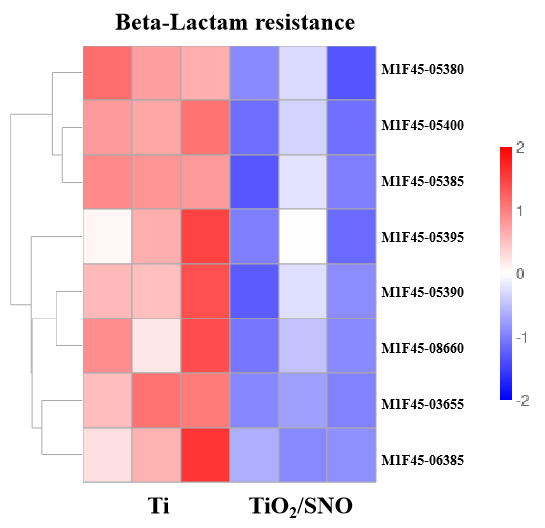


Figure S12. Heat map of Beta-Lactam resistance genes.


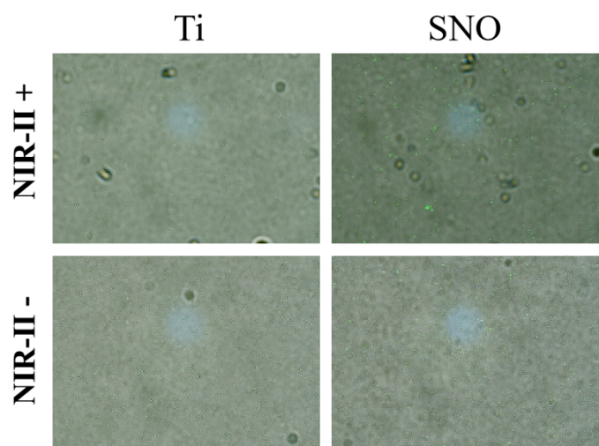


Figure S13. Fluorescence staining of macrophages phagocytosing bacteria.


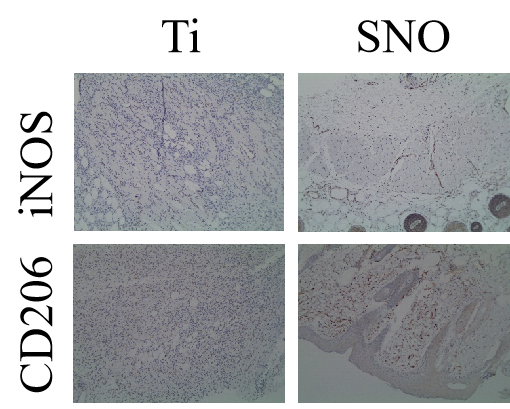


Figure S14. iNOS and CD206 immunohistochemistry of tissues surrounding the implants after phototherapy for 4 hours.


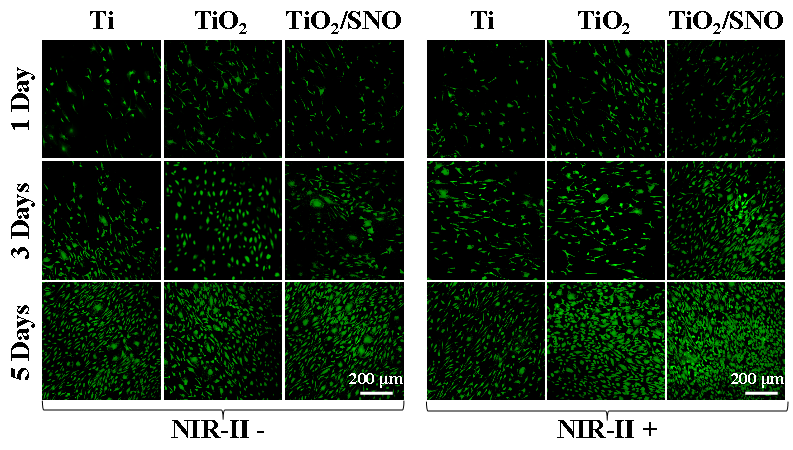


Figure S15. Live/dead (green/red) fluorescence staining images of HUVECs.


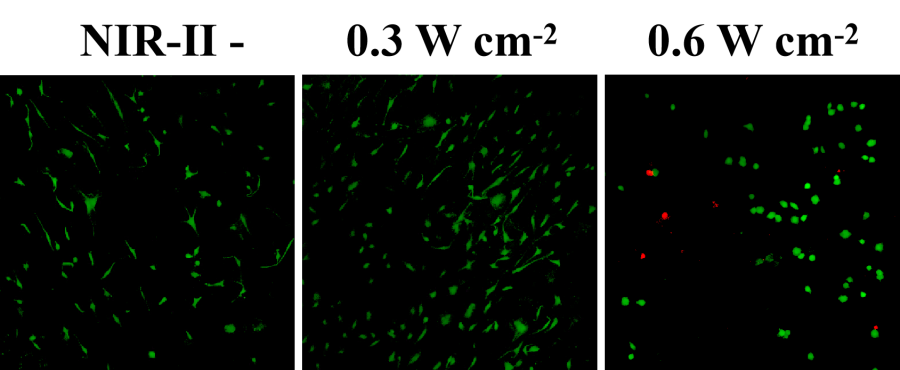


Figure S16. Live/dead (green/red) fluorescence staining images of HUVECs on TiO_2_/SNO irradiated with the 1,060 nm laser (0.3 W cm^-2^ and 0.6 W cm^-2^).


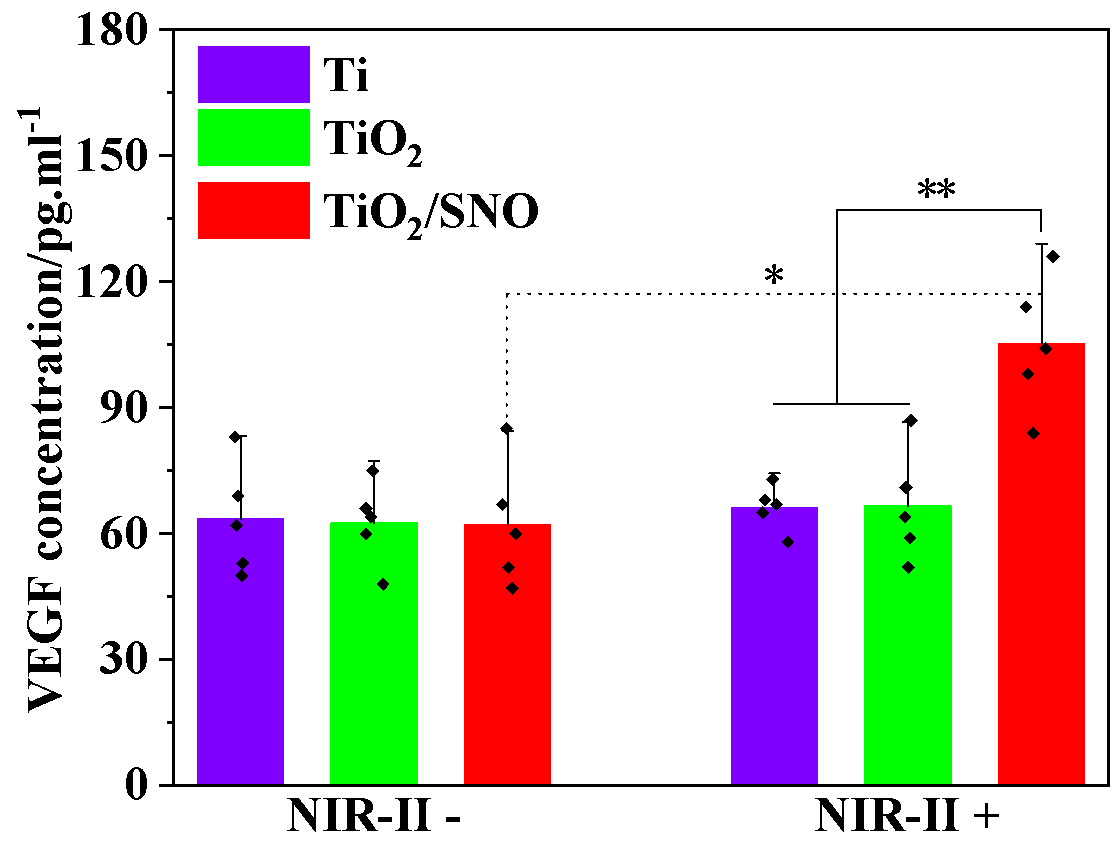


Figure S17. VEGF secretion from HUVECs.

Figure S18. Live/dead (green/red) fluorescence staining images of BMSCs on TiO_2_/SNO irradiated with the 1,060 nm laser (0.3 W cm^-2^ and 0.6 W cm^-2^).

Figure S19. Weight changes of rats after surgery.


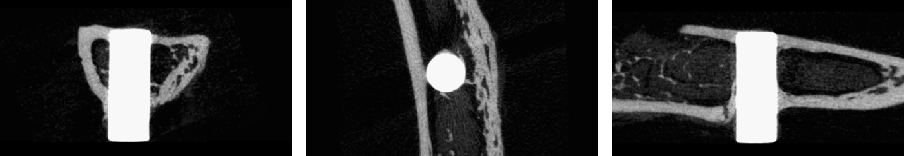


Figure S20. 2D images of the TiO_2_/SNO implant reconstructed by micro-CT.


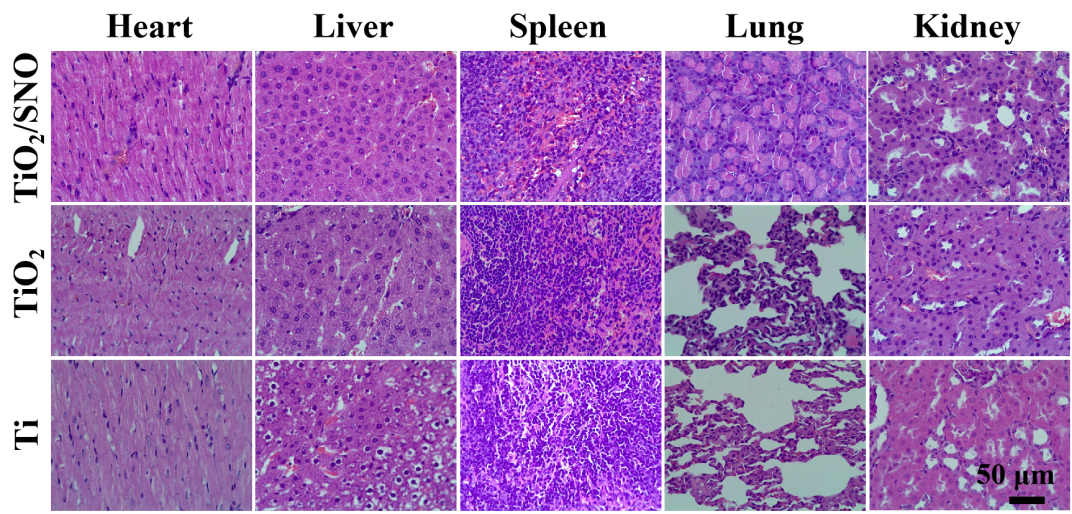


Figure S21. Histological analysis of major organs, including liver, spleen, kidney, heart, and lung, by H&E staining.

**Table S1.** Primers of target genes.

| Genes | Gene forward primer sequence (5’–3’) | Reverse primer sequence (5’–3’) |
| --- | --- | --- |
| murB | CGCACCACCAAACTCCCT | CCAGACGCCAGAACATCG |
| femA | AAGCGAGATAACTTACAAC | AAAGAAGAAACCAGCAGA |
| sgtB | TCTGGAGGATACGTTGCT | ATGGACGATGTGAAATGAG |
| 16s rRNA | ACAATACAAAGGGCAGCGAAAC | CGATACGGCTACCTTGTTACGAC |

**Table S2.** Primers for the target genes.

| Genes | Gene forward primer sequence (5’–3’) | Reverse primer sequence (5’–3’) |
| --- | --- | --- |
| GAPDH | GGTTGTCTCCTGCGACTTCA | TGGTCCAGGGTTTCTTACTCC |
| ALP | GCCCTCCAGA TCCTGACCAA | GCAGAGCCTGCTGGTCCTTA |
| OPN | ATCTCCTTGCGCCACAGAATGC | ATCTCCTTGCGCCACAGAATGC |
| RUNX2 | AACCCACGAATGCACTACCCA | GGAACTGATAGGATGCTGACGAAG |

**Table S3**. The relative chemical composition (at.%) of the surface elements on TiO_2_, TiO_2_/APTES, TiO_2_/SNO detected by XPS.

| Samples | Ti 2p (%) | C 1s (%) | N 1s (%) | O 1s (%) | Si 2p (%) | S 2p (%) |
| --- | --- | --- | --- | --- | --- | --- |
| TiO_2_ | 23.78 | 16.7 | 5.07 | 52.92 | 0 | 0 |
| TiO_2_/APTES | 15.07 | 30.26 | 7.85 | 38.28 | 4.83 | 0 |
| TiO_2_/SNO | 10.08 | 42.78 | 14.49 | 24.49 | 2.06 | 3.14 |
